# Development of the “POP” scoring system for predicting obstetric and gynecological diseases in the emergency department: a retrospective cohort study

**DOI:** 10.1186/s12873-020-00332-z

**Published:** 2020-05-06

**Authors:** Asami Okada, Yohei Okada, Hiroyuki Fujita, Ryoji Iiduka

**Affiliations:** 1 Department of Emergency Medicine and Critical Care, Japanese Red Cross Society Kyoto Daini Hospital, 602-8026, Haruobicho 355-5, Kamigyo, Kyoto, Japan; 2 Department of Obstetrics and Gynecology, Japanese Red Cross Society Kyoto Daini Hospital, Kyoto, Japan; 3 Department of Preventive Services, School of Public Health, Kyoto University, Kyoto, Japan; 4 Department of Primary Care and Emergency Medicine, Graduate School of Medicine, Kyoto University, Kyoto, Japan

**Keywords:** Abdominal pain, Emergency medicine, Prediction model, Gynecologic emergencies, Screening

## Abstract

**Background:**

Obstetric and gynecological (OBGY) diseases are among the most important differential diagnoses for young women with acute abdominal pain. However, there are few established clinical prediction rules for screening OBGY diseases in emergency departments (EDs). This study aimed to develop a prediction model for diagnosing OBGY diseases in the ED.

**Methods:**

This single-center retrospective cohort study included female patients with acute abdominal pain who presented to our ED. We developed a logistic regression model for predicting OBGY diseases and assessed its diagnostic ability. This study included young female patients aged between 16 and 49 years who had abdominal pain and were examined at the ED between April 2017 and March 2018. Trauma patients and patients who were referred from other hospitals or from the OBGY department of our hospital were excluded.

**Results:**

Out of 27,991 patients, 740 were included. Sixty-five patients were diagnosed with OBGY diseases (8.8%). The “POP” scoring system (past history of OBGY diseases + 1, no other symptoms + 1, and peritoneal irritation signs + 1) was developed. Cut-off values set between 0 and 1 points, sensitivity at 0.97, specificity at 0.39, and negative likelihood ratio (LR-) of 0.1 (95% CI: 0.02–0.31) were considered to rule-out, while cut-off values set between 2 and 3 points, sensitivity at 0.23 (95% CI 0.13–0.33), specificity at 0.99 (95% CI 0.98–1.00), and positive likelihood ratio (LR+) of 17.30 (95% CI: 7.88–37.99) were considered to rule-in.

**Conclusions:**

Our “POP” scoring system may be useful for screening OBGY diseases in the ED. Further research is necessary to assess the predictive performance and external validity of different data sets.

## Background

Acute abdominal pain is one of the most common severe presentations in emergency departments (EDs), and its differential diagnosis includes a very broad range of possible etiologies. Thus, a systematic diagnostic procedure is necessary in making an appropriate diagnosis. In young women with abdominal pain, obstetric and gynecological (OBGY) diseases, such as ectopic pregnancy, represent two of the most important differential diagnoses. This is because a delayed diagnosis can be life-threatening, affect the reproductive function, and decline the quality of life [1, 2].

Generally, a diagnosis of OBGY diseases requires vaginal examinations and pelvic or transvaginal ultrasonography by trained specialists such as OBGY physicians [3, 4]. However, their availability is limited in the ED in Japan. Therefore, an easy screening tool is necessary to estimate the possibility of OBGY disease for appropriate consultations with OBGY physicians. Nevertheless, there are few established clinical prediction rules for screening emergency OBGY disease in the ED.

Therefore, this study aimed to develop and validate a prediction model for the diagnosis of OBGY diseases in the ED.

## Methods

This study is a retrospective cohort study to develop a prediction model, which complied with the TRIPOD statement (Transparent Reporting of a Multivariable Prediction Model for Individual Prognosis or Diagnosis) regarding the reporting of the study’s methods and results [5]. This study was approved by the Clinical Research Ethics Committee of Kyoto Daini Red Cross Hospital (Approval ID No. 2018–08). The ethics committee waived the requirement for informed consent because of the anonymous nature of the data.

### Data source and settings

We obtained the clinical data by an electronic chart review from the Japanese Red Cross Society Kyoto Daini Hospital Tertiary Critical Care Center in Kyoto City, Japan. Kyoto City is an urban area that has a population of approximately 1.5 million, and the total number of ambulance calls per year is approximately 80,000 cases in the entire city [6]. There are four critical care tertiary centers in Kyoto City, and this 672-bed hospital is one of those located at the center of Kyoto City. This hospital provides primary to tertiary emergency care for any type of emergency cases such as severe trauma, cardiac arrest, and stroke. There were 7679 cases where the patients arrived by ambulances and 20,312 cases where the patients visited by walk-in to our ED in 2017. Hence, consultations with an OBGY physician on duty and an emergency surgeon are always available if necessary.

### Study population

The study population included young female patients aged between 16 and 49 years old, who had abdominal pain and were examined at the ED between April 2017 and March 2018. We selected patients from among those who met the inclusion criteria by reviewing their chief complaints and medical history of all electronic charts of young female patients who presented to the ED. Trauma patients and patients who were referred from another hospital or from the OBGY department of our hospital were excluded.

### Data collection and patient outcomes

We collected the following clinical data upon ED admission through the electronic chart review: age, time of hospital arrival, mode of ED visit (walk-in or ambulance), medical past history of OBGY disease, the symptoms (fever, digestive symptoms [e.g. vomiting, diarrhea]), atypical genital bleeding, and signs of peritoneal irritation upon physical examination. We defined medical history of OBGY disease as previous surgery due to gynecologic diseases such as ovarian, uterine, pelvic inflammatory, or sexually transmitted diseases. We also defined peritoneal irritation signs as muscular defense, guarding, rigidity, rebound tenderness, percussion tenderness, or heel drop test positive, based on the Japanese practice guideline for primary care of acute abdomen 2015 [7]. We also collected data on final diagnosis at hospital discharge based on the International Statistical Classification of Diseases and Related Health Problems (ICD-10).

The primary outcome of interest was defined as final diagnosis of OBGY disease except for menstrual pain, which was determined by an OBGY or ED physician.

### Prognostic variable selection, handling missing data, and sample size estimation

Based on previous studies [1, 8], our experience, and expert opinion we selected three variables (past history of OBGY disease, no other symptom, and peritoneal irritation sign) as potential predictors of diagnosis of OBGY disease.

Missing data were categorized as “unknown” because unmeasured values might be informative in clinical settings. For sample size estimation, there are no generally accepted approaches to estimate the sample size criteria for deriving risk prediction models. Although we know that it is controversial [9], we took account of the idea at 10 events per variable may be necessary for deriving a logistic model based on some empirical investigation [10]. There, we estimated that our study had an adequate sample size to develop the prediction model.

### Statistical analysis

We described the patients’ characteristics. We calculated each variable’s ß coefficient and crude odds ratios (ORs) with 95% confidence intervals (CIs) using univariable logistic regression models. Furthermore, we identified the adjusted OR with 95% CI using the multivariable logistic model including all predictors. The model’s performance was evaluated based on the C-statistics, the calibration intercept and slope, and the Brier score [10]. As interval validation, optimization of the model was estimated by a bootstrapping procedure using 1000 samples with replacement from the original sample [10, 11]. Finally, we set the clinically useful simplified screening system using a simple integer score based on each variable’s ß coefficient. The diagnostic abilities (sensitivity, specificity, positive likelihood ratio [LR+], and negative likelihood ratio [LR-]) of each score were calculated. Generally, high LR+ (i.e., ≥10) or low LR- (i.e., ≤ 0.1) was considered strong evidence to rule-in or rule-out the target condition [12]. Thus, we set the cut-off point to rule-in or rule-out OBGY diseases based on the calculated LR. The calibration performance of risk stratification was graphically evaluated in terms of the relationship between the predicted probability and observed proportion of the OBGY disease diagnosis. All statistical results were considered significant at two-sided *P* values of < 0.05. Statistical analyses were performed using JMP Pro® 14 software (SAS Institute Inc., Cary, NC) and R software (version 1.1.456; R Studio Inc.) with the “rms” package [13].

## Results

### Patient characteristics

**Fig. 1:**
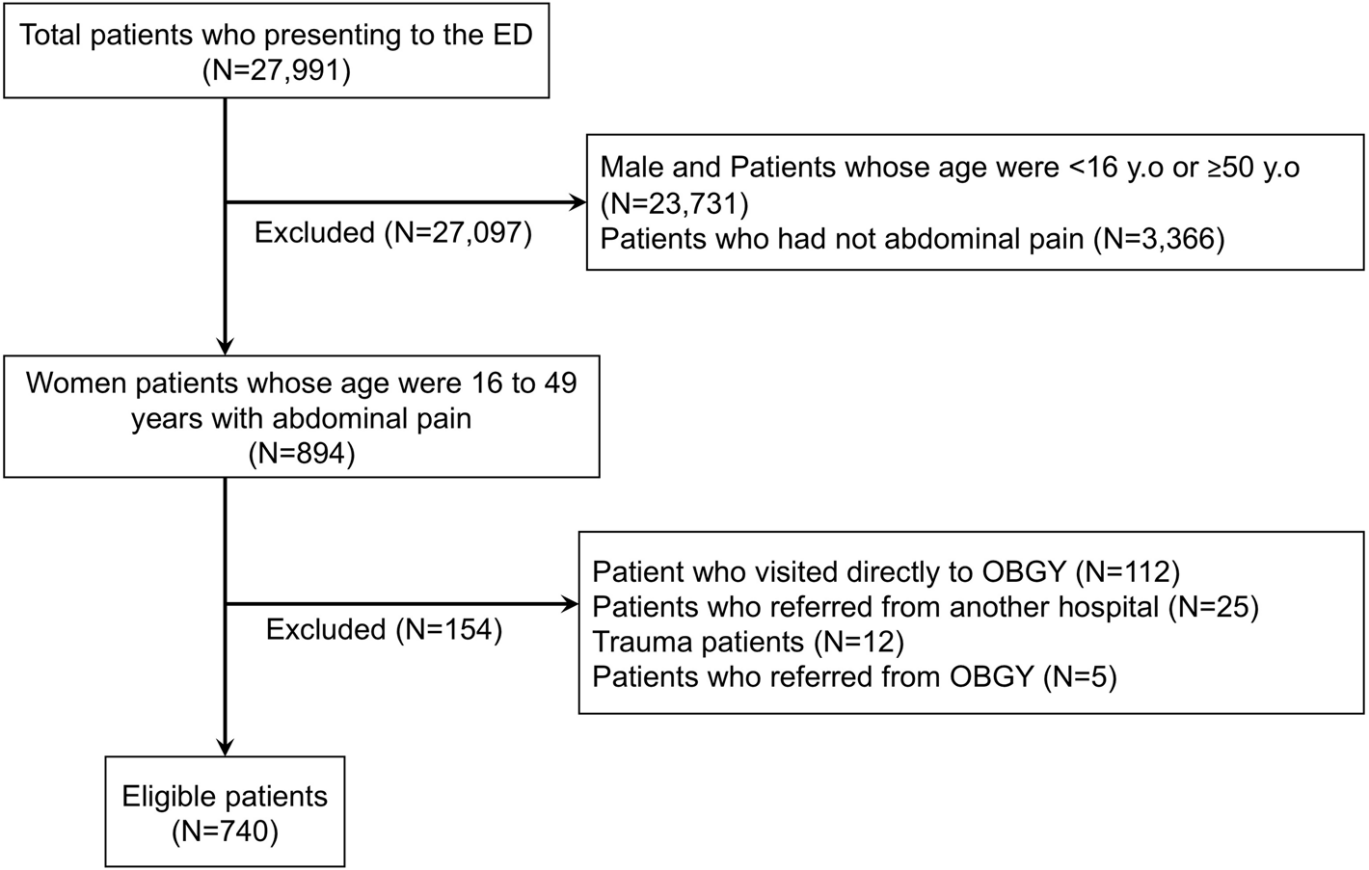
Flow chart of patient selection. ED: emergency department, OBGY: obstetric and gynecological

Among the 27,991 patients presented to the ED, 894 young female patients had acute abdominal pain. We excluded 112 patients who directly consulted an OBGY physician, 25 patients who were referred from another hospital, 12 patients with trauma, and 5 patients who were referred from the OBGY department in our hospital. Finally, 740 patients were included for the analysis (Fig. 1). Out of these patients, except for menstrual pain (*N* = 51), 65 patients were diagnosed with OBGY disease (8.8%). The characteristics of the patients are shown in Table 1. Details of OBGY diseases and all diseases are shown in Table 2 and supplementary file 1.

**Table 1:** Characteristics of the study participants

Parameters	Total (***N*** = 740)	Final diagnosis				
		OBGY diseases (***N*** = 65)	Menstrual pain (N = 51)	Digestive diseases (***N*** = 489)	Urological diseases (***N*** = 31)	Others (***N*** = 104)
**Age, median, (IQR)**	30 (23.0–39.0)	30 (22.0–40.5)	24 (20.0–32.0)	30 (23.0–39.0)	33 (24.0–41.0)	31 (23.0–39.75)
**< 20 years, n, (%)**	76 (10.3%)	3 (4.6%)	6 (11.8%)	56 (11.5%)	0 (0.0%)	11 (10.6%)
**20–29**	278 (37.6%)	25 (38.5)	28 (54.9%)	176 (36.0%)	12 (38.7%)	37 (35.6%)
**30–39**	205 (27.7%)	19 (29.2%)	9 (17.6%)	136 (27.8%)	11 (35.5%)	30 (28.8%)
**> 40**	181 (24.5%)	18 (27.7%)	8 (15.7%)	121 (24.7%)	8 (25.8%)	26 (25.0%)
**How to visit**						
**Walk in, n, (%)**	591 (79.9%)	47 (72.3%)	16 (31.4%)	412 (84.3%)	26 (83.9%)	14 (13.5%)
**Ambulance**	149 (20.1%)	18 (27.7%)	35 (68.6%)	77 (15.7%)	5 (16.1%)	90 (86.5%)
**Past medical history of OBGY diseases**						
**Yes, n, (%)**	153 (20.7%)	32 (49.2%)	14 (27.5%)	84 (17.2%)	4 (12.9%)	19 (18.3%)
**No**	587 (79.3%)	33 (50.8%)	37 (72.5%)	405 (82.8%)	27 (87.1%)	85 (81.7%)
**Other symptoms**						
**Digestive symptoms, n, (%)**	292 (39.5%)	7 (10.8%)	5 (9.8%)	250 (51.1%)	8 (25.8%)	22 (21.2%)
**Fever**	32 (4.3%)	2 (3.1%)	0 (0.0%)	26 (5.3%)	1 (3.2%)	3 (2.9%)
**Nothing**	386 (52.2%)	56 (86.2%)	45 (88.2%)	203 (41.5%)	13 (41.9%)	68 (65.4%)
**Other**	30 (4.1%)	0 (0.0%)	1 (2.0%)	10 (2.0%)	9 (29.0%)	11 (10.6%)
**Atypical genital bleeding, n, (%)**	2 (0.3%)	1 (1.5%)	0 (0.0%)	0 (0.0%)	0 (0.0%)	1 (1.0%)
**Peritoneal irritation signs, n, (%)**	119 (16.1%)	30 (46.2%)	2 (3.9%)	79 (16.2%)	2 (6.5%)	6 (5.8%)
**Admission, n, (%)**	81 (10.9%)	22 (33.8%)	0 (0.0%)	56 (11.5%)	1 (3.2%)	2 (1.9%)
**Emergency operation, n, (%)**	38 (5.1%)	14 (21.5%)	0 (0.0%)	24 (4.9%)	0 (0.0%)	0 (0.0%)

*IQR* Interquartile range, *OBGY* Obstetric and gynecological

**Table 2:** Details of OBGY disease

Details of OBGY diseases	N = 65
Rupture of ovarian tumor	12 (18.5%)
PID	12 (18.5%)
Ovarian bleeding	8 (12.3%)
Adnexal torsion	7 (10.8%)
Uterine myoma	6 (9.2%)
Ovulation pain	5 (7.7%)
Ectopic pregnancy	3 (4.6%)
Malignant tumor	1 (1.5%)
Abortion	1 (1.5%)
Other	10 (15.4%)

*OBGY* Obstetric and gynecological, *PID* Pelvic inflammatory disease

### Performance and internal validation of the model

The variables’ ß coefficient, crude ORs with 95% CI, adjusted ORs with 95% CI, and formula for predicted diagnosis of OBGY disease are shown in the Table 3. As an internal validation, we also described the bias-corrected model performance using bootstrap procedure in the supplementary file 2. It indicated that the risk of bias by overfitting was estimated as low.

**Table 3:** β coefficient, CORs, and AORs for each predictor

Predictors	β coefficient	CORs (95% CI)	β coefficient	AORs (95% CI)
**Past history of OBGY disease**	0.75	4.44 (2.63–7.50)	0.65	3.69 (2.11–6.47)
**Noother symptoms**	0.88	5.78 (2.90–11.54)	0.80	4.95 (2.43–10.10)
**Peritoneal irritation signs**	0.87	5.64 (3.30–9.65)	0.80	4.96 (2.80–8.79)

*COR* Crude odds ratio, *AOR* Adjusted odds ratio, *CI* Confidence intervals, *OBGY* Obstetric and gynecological

**Fig. 2:**
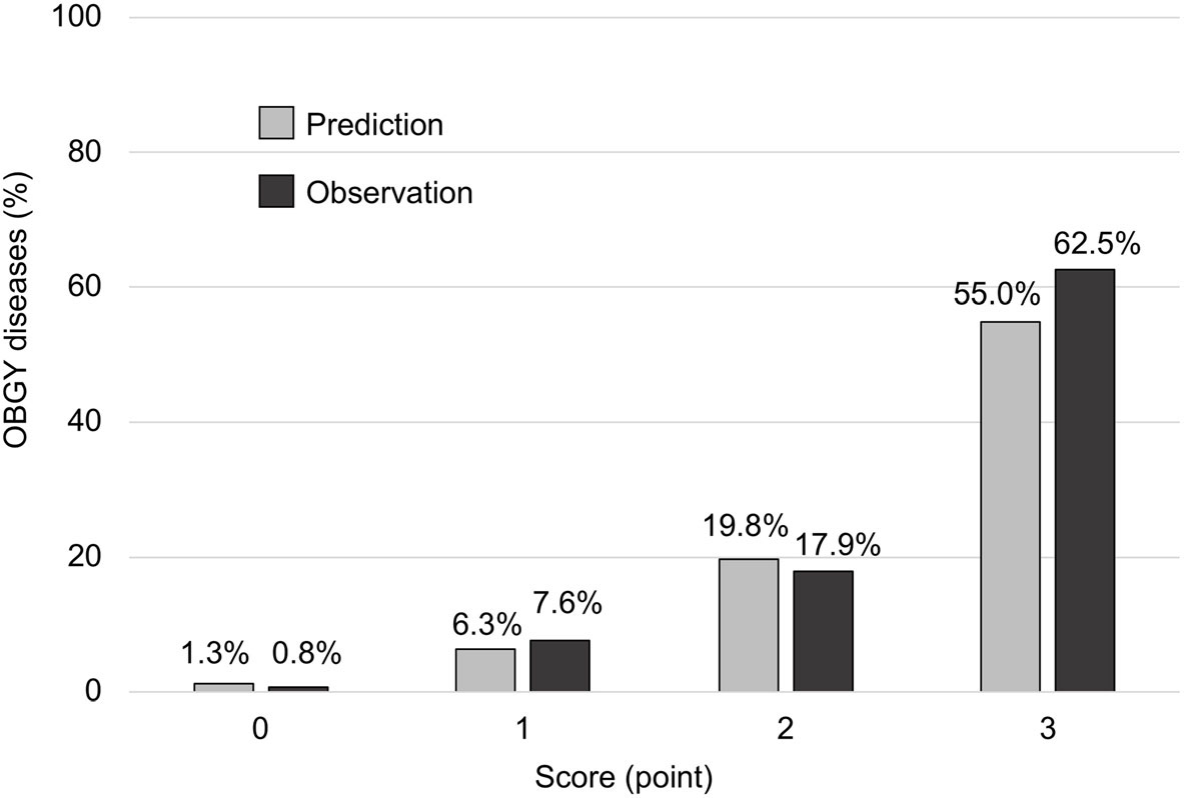
Calibration of prediction and observation. Predicted probability and observed proportion of OBGY diseases based on the POP scoring system. The mean predicted probability is shown by for the sums of the scores in each cohort. Observation reflected the observed proportion of diagnosed OBGY diseases. The predictions were well calibrated with the observations. The POP scoring system provided a simple and rapid prediction of OBGY diseases in ED. OBGY: obstetric and gynecological.

As rounded β coefficient of the predictors was almost the same, we set identical weights on the predictors to make it easy to remember in clinical settings and created a prediction scoring system, the POP score (Past history of OBGY disease: + 1 point, no Other symptom: + 1 point, and Peritoneal irritation sign: + 1 point). The C-statistics of this score was 0.794 (95% CI: 0.74–0.84). Particularly, if the cut-off values were set between 0 and 1 point, sensitivity was 0.97 (95% CI: 0.92–1.01), specificity was 0.39 (95% CI: 0.35–0.43), LR- was 0.08(95% CI: 0.02–0.31), and negative predictive value was 0.99 (95% CI: 0.98–1.00); thus, this was suitable to rule out OBGY disease. Further, if the cut-off values were set between 2 and 3 points, sensitivity was 0.23 (95% CI: 0.13–0.33), specificity was 0.99 (95% CI: 0.98–1.00), LR+ was 17.30 (95% CI: 7.88–37.99), and the positive predictive value was 0.63 (95% CI: 0.43–0.82); thus, this cut-off was appropriate to rule-in OBGY disease (Table 4). Additionally, graphical evaluation of the scoring system revealed good calibration between prediction and observation (Fig. 2).

**Table 4:** Diagnostic ability for each cut-off

Score cut off	TP	FP	TN	FN	Sensitivity (95%CI)	Specificity (95%CI)	LR+ (95%CI)	LR- (95%CI)	PPV (95%CI)	NPV (95%CI)
**3/2**	15	9	666	50	0.23 (0.13–0.33)	0.99 (0.98–1.00)	17.30 (7.88–37.99)	0.78 (0.68–0.89)	0.63 (0.43–0.82)	0.93 (0.91–0.95)
**2/1**	39	119	556	26	0.60 (0.48–0.72)	0.82 (0.79–0.85)	3.40 (2.63–4.40)	0.49 (0.36–0.66)	0.25 (0.18–0.31)	0.96 (0.94–0.97)
**1/0**	63	411	264	2	0.97 (0.92–1.00)	0.39 (0.35–0.43)	1.59 (1.48–1.71)	0.08 (0.02–0.31)	0.13 (0.10–0.16)	0.99 (0.98–1.00)

*LR*+ Positive likelihood ratio, *LR*- Negative likelihood ratio, *TP* True positive, *FP* False positive, *TN* True negative, *FN* False negative, *PPV* Positive predictive value, *NPV* Negative predictive value, *CI* Confidence interval

## Discussion

### Key observations

The present study showed that the “POP” scoring system (Past history of OBGY, no Other symptoms, and Peritoneal irritation sign) had a good screening ability for OBGY disease, with good discrimination and calibration with internal validation in the ED setting.

### Previous literature and the present study’s strengths

Compared to previous studies, our study has some strengths for generalizability. A previous prospective multicenter study on five OBGY departments in Paris (*N* = 516) developed and validated a clinical prediction rule for identifying life-threatening diseases (e.g. ectopic pregnancy, adnexal torsion or tubo-ovarian abscess which can lead to hemodynamic instability, organ failures, severe morbidity, and death) in gynecological emergency rooms in patients with acute pelvic pain. Vomiting, sudden onset of fever, and pain from palpation are significantly associated with life-threatening diseases [1]. However, the setting of this previous study was on gynecological emergency, which was substantially different from the primary care or general EDs. In addition, this previous study did not include various types of diseases such as digestive or urological diseases. Thus, its generalizability may be limited (spectrum bias). Conversely, our study setting was general ED in an urban area. Therefore, our study had the strength in terms of generalizability as compared to other ED settings.

Other previous prospective studies in the United States developed and validated a prediction model for ectopic pregnancy in the ED [8]. In this previous study, patients were limited to women with early pregnancy who visited the ED, and its predictors included cervical motion tenderness and fetal heart rate. For non-gynecologist physicians, the opportunity to perform vaginal examinations or transvaginal ultrasonography is extremely limited in Japan. Thus, this previous model to predict ectopic pregnancy also cannot be applied to general EDs. Accordingly, we believe that our prediction rule may be more reliable for diagnosing or excluding OBGY diseases in general ED.

### Interpretation

We suggested possible explanations of this prediction model. The present study evaluated clinically relevant variables that can be summarized as “POP” (past history of OBGY disease, other symptoms, and peritoneal irritation sign). In terms of the past history of OBGY diseases, it is reported that ovarian tumor rupture and adnexal torsion are likely to occur and recur in patients with a history of ovarian tumor [2, 14, 15]. Accordingly, past history of OBGY diseases is an important clinical information for prediction. Moreover, previous studies reported that vomiting was associated with tubal rupture and adnexal torsion [16, 17]. However, the previous study’s population included patients who were only diagnosed with OBGY diseases. Conversely, most patients in our study (489/740: 66%) were diagnosed with digestive diseases; half of them (250/489: 51%) complained of digestive symptoms such as vomiting, while only 12% (8/65) with OBGY diseases had vomiting. Hence, it may be reasonable that no other symptom was more associated with OBGY diseases than other cases especially those related to digestive disease in general ED. Moreover, in terms of fever, there was no association between fever and OBGY diseases [16]. On peritoneal irritation signs, most patients with ectopic pregnancy had abdominal peritoneal signs [8]. Thus, we assumed that ovarian bleeding and ectopic pregnancy cause bleeding in the pelvic cavity, and pelvic inflammatory disease causes localized inflammation in the pelvis. Similarly, we found that most patients hospitalized for OBGY disease or those who underwent emergency surgery for OBGY disease also had peritoneal irritation sign. Atypical genital bleeding can be expected to be associated with OBGY diseases. However, in this study, there were only 2 cases out of 740 cases with an atypical genital bleeding. Therefore, the association between atypical genital bleeding and OBGY diseases was unknown in our study.

Hence, it is reasonable that these results can be reliable and valid clinical predictors of OBGY diseases.

### Clinical implications

The clinical implications of this study are that OBGY diseases can diagnosed or excluded based on this simple scoring system. When score cut-off was set at 0/1 point, the negative likelihood ratio was 0.1 in our findings, which is useful to rule out OBGY diseases. If the prior probability (8.8%) was the same as in our setting, the posterior probability decreased to 1.3% when the score was 0. As an expected advantage of easy screening to exclude OBGY disease diagnosis, we presumed that there would be decrease in unnecessary consultation and number of transfers from hospitals without obstetricians and gynecologists, thereby reducing specialist physicians’ workload. When score cut-off was set at 2/3 points, the positive likelihood ratio was 17.3. The posterior probability increased to 55% in the abovementioned setting, when the score was 3. It may be useful for rule-in, leading to appropriate consultation. We suggest consultation with gynecologists if the POP score is 3 points. Meanwhile, if the POP score was 1 or 2 points, we considered evaluating the results from other additional tests (e.g., blood test, transabdominal ultrasonography, and computed tomography). Accordingly, the results of our study indicate that the POP score may be useful to rule-out or rule-in OBGY disease in an ED setting.

### Limitations

Our study has several limitations. Firstly, this is a retrospective study based on chart review, wherein the validity of the diagnosis, measurement factors, and the missed diagnosis might have led to information bias. Secondly, direct visitation to OBGY department may have led to selection bias. Thirdly, we could not assess the external validation as our study was conducted in a single center, with a relatively small sample size. Despite using the bootstrap procedure, our results indicated a low risk of bias by overfitting. Thus, further research is necessary to evaluate the external validation and applicability to other areas and in multi-centers.

## Conclusion

We showed that the “POP” scoring system had good discrimination and calibration for the diagnosis of OBGY diseases in young female patients with abdominal pain who presented to the ED. Further research is necessary to assess the predictive performance and external validity of different data sets.

## Supplementary information

**Additional file 1: Table S1** Detail of all diseases. **Table S2**. Prediction performance corrected by bootstrap

## Data Availability

Data sharing is not applicable to this article as ethics committee did not approve it.

## Abbreviations

OBGY: Obstetric and gynecological
ED: Emergency department
OR: Odds ratio
LR: Likelihood ratio
