# Supplementary material for: Development of the “POP” scoring system for predicting obstetric and gynecological diseases in the emergency department: a retrospective cohort study
Source: BMC Emerg Med. 2020 May 6;20:35. doi: 10.1186/s12873-020-00332-z (PMC7203896; doi:10.1186/s12873-020-00332-z)
Supplement: Supplementary file 1 — Additional file 1: Table S1 Detail of all diseases. Table S2. Prediction performance corrected by bootstrap [file 12873_2020_332_MOESM1_ESM.docx]

**Supplementary File: Table 1. Detail of all diseases.**

| **Detail diagnosis** | **N=740** |
| --- | --- |
| Acute enteritis | 318(43.0%) |
| Unknown | 95(12.8%) |
| Menstrual pain | 51(6.9%) |
| Appendicitis | 35(4.7%) |
| Constipation | 29(3.9%) |
| GDU | 28(3.9%) |
| Diverticulitis, diverticular bleeding | 24(3.2%) |
| Other of gastroenterology | 19(2.6%) |
| Urinary stone | 13(1.8%) |
| Rupture of ovarian tumor | 12(1.6%) |
| PID | 12(1.6%) |
| Cholelithiasis | 11(1.5%) |
| Ileus | 11(1.5%) |
| Other of OBGY | 10(1.4%) |
| Pyelonephritis | 9(1.2%) |
| Other of urology | 9(1.2%) |
| Other | 9(1.2%) |
| Ischemic enteritis | 8(1.1%) |
| Ovarian bleeding | 8(1.1%) |
| Adnexal torsion | 7(0.9%) |
| Uterine myoma | 6(0.8%) |
| Ovulation pain | 5(0.7%) |
| Intestinal perforation, panperitonitis | 4(0.5%) |
| Ectopic pregnancy | 3(0.4%) |
| Pancreatitis | 2(0.3%) |
| Malignant tumor | 1(0.1%) |
| Abortion | 1(0.1%) |
| GDU: gastroduodenal ulcer, PID: pelvic inflammatory disease, OBGY: obstetrics and gynecology. | |

**Supplementary File: Table 2. Prediction performance corrected by bootstrap**

| Index | Original | Optimism | Index. corrected |
| --- | --- | --- | --- |
| c-statistics | 0.803 | 0.014 | 0.796 |
| R^2^ | 0.244 | 0.012 | 0.231 |
| Intercept | 0.000 | 0.024 | -0.024 |
| Slope | 1.000 | 0.024 | 0.976 |
| Brier score | 0.068 | 0.002 | 0.069 |
